# Supplementary material for: Empirical methods for controlling false positives and estimating confidence in ChIP-Seq peaks
Source: BMC Bioinformatics. 2008 Dec 5;9:523. doi: 10.1186/1471-2105-9-523 (PMC2628906; doi:10.1186/1471-2105-9-523)
Supplement: Additional File 1 — A variety of html documents from the USeq web site detailing the available applications, their best usage, output file type descriptions, command line menus, etc. [file 1471-2105-9-523-S1.zip › USeqUserGuides/JavaDoc.zip!/JavaDoc/bioroot/UserBean.html]

UserBean


|  |  |  |
| --- | --- | --- |
| **Overview** \| **Package** \| **Class** \| **Use** \| **Tree** \| **Deprecated** \| **Index** \| **Help** | |  |
| **PREV CLASS**   **NEXT CLASS** | **FRAMES**    **NO FRAMES**     **All Classes** |  |
| SUMMARY: NESTED \| FIELD \| CONSTR \| METHOD | DETAIL: FIELD \| CONSTR \| METHOD |  |


---


## bioroot Class UserBean

```
java.lang.Object
  bioroot.UserBean
```

---

``` public class UserBean extends java.lang.Object ```

User information.

---

**Constructor Summary**

`UserBean()`

`UserBean(DBUtil db,
LoginBean loginBean)`


| **Method Summary** | |
| --- | --- |
| `Preference` | `getAntibodyPreference()` |
| `java.lang.String` | `getAntibodyPrefs()` |
| `java.lang.String` | `getEditHistory()` |
| `java.lang.String` | `getEmail()` |
| `int` | `getFailedLogins()` |
| `java.lang.String` | `getFirstName()` |
| `int` | `getId()` |
| `java.lang.String` | `getLabGroup()` |
| `int` | `getLabGroupId()` |
| `int` | `getLabGroupSchedule(DBUtil bioRoot)` |
| `java.lang.String` | `getLastLogin()` |
| `java.lang.String` | `getLastName()` |
| `java.lang.String` | `getLoginName()` |
| `Preference` | `getOligoPreference()` |
| `java.lang.String` | `getOligoPrefs()` |
| `Organization` | `getOrganization()` |
| `java.lang.String` | `getPassword()` |
| `java.lang.String` | `getPhone()` |
| `Preference` | `getPlasmidPreference()` |
| `java.lang.String` | `getPlasmidPrefs()` |
| `java.lang.String` | `getRemoteHost()` |
| `StrainPreference` | `getStrainPreference()` |
| `java.lang.String` | `getStrainPrefs()` |
| `java.lang.String` | `getType()` |
| `boolean` | `isGuest()` |
| `boolean` | `isGuestOrFriend()` |
| `boolean` | `isSuperUser()` |
| `void` | `setAntibodyPreference(Preference antibodyPreference)` |
| `void` | `setEditHistory(java.lang.String string)` |
| `void` | `setEmail(java.lang.String string)` |
| `void` | `setFailedLogins(int i)` |
| `void` | `setFirstName(java.lang.String string)` |
| `void` | `setId(int i)` |
| `void` | `setLabGroup(java.lang.String string)` |
| `void` | `setLabGroupId(int labGroupId)` |
| `void` | `setLastLogin(java.lang.String string)` |
| `void` | `setLastName(java.lang.String string)` |
| `void` | `setLoginName(java.lang.String string)` |
| `void` | `setOligoPreference(Preference oligoPreference)` |
| `void` | `setPassword(java.lang.String string)` |
| `void` | `setPhone(java.lang.String string)` |
| `void` | `setPlasmidPreference(Preference plasmidPreference)` |
| `void` | `setRemoteHost(java.lang.String string)` |
| `void` | `setStrainPreference(StrainPreference strainPreference)` |
| `void` | `setType(java.lang.String string)` |

**Methods inherited from class java.lang.Object**

`equals, getClass, hashCode, notify, notifyAll, toString, wait, wait, wait`

**Constructor Detail**

### UserBean

```
public UserBean()
```

---


### UserBean

```
public UserBean(DBUtil db,
                LoginBean loginBean)
```


**Method Detail**

### isGuest

```
public boolean isGuest()
```

---


### isGuestOrFriend

```
public boolean isGuestOrFriend()
```

---


### getLabGroupSchedule

```
public int getLabGroupSchedule(DBUtil bioRoot)
```

---


### getEmail

```
public java.lang.String getEmail()
```

---


### getFirstName

```
public java.lang.String getFirstName()
```

---


### getLabGroup

```
public java.lang.String getLabGroup()
```

---


### getLastLogin

```
public java.lang.String getLastLogin()
```

---


### getLastName

```
public java.lang.String getLastName()
```

---


### getRemoteHost

```
public java.lang.String getRemoteHost()
```

---


### getType

```
public java.lang.String getType()
```

---


### getPhone

```
public java.lang.String getPhone()
```

---


### getId

```
public int getId()
```

---


### setEmail

```
public void setEmail(java.lang.String string)
```

---


### setFirstName

```
public void setFirstName(java.lang.String string)
```

---


### setLabGroup

```
public void setLabGroup(java.lang.String string)
```

---


### setLastName

```
public void setLastName(java.lang.String string)
```

---


### setPhone

```
public void setPhone(java.lang.String string)
```

---


### setType

```
public void setType(java.lang.String string)
```

---


### isSuperUser

```
public boolean isSuperUser()
```

---


### getOligoPrefs

```
public java.lang.String getOligoPrefs()
```

---


### getFailedLogins

```
public int getFailedLogins()
```

---


### getEditHistory

```
public java.lang.String getEditHistory()
```

---


### getLoginName

```
public java.lang.String getLoginName()
```

---


### getPassword

```
public java.lang.String getPassword()
```

---


### setEditHistory

```
public void setEditHistory(java.lang.String string)
```

---


### setFailedLogins

```
public void setFailedLogins(int i)
```

---


### setId

```
public void setId(int i)
```

---


### setLastLogin

```
public void setLastLogin(java.lang.String string)
```

---


### setLoginName

```
public void setLoginName(java.lang.String string)
```

---


### setPassword

```
public void setPassword(java.lang.String string)
```

---


### setRemoteHost

```
public void setRemoteHost(java.lang.String string)
```

---


### getLabGroupId

```
public int getLabGroupId()
```

---


### setLabGroupId

```
public void setLabGroupId(int labGroupId)
```

---


### getOligoPreference

```
public Preference getOligoPreference()
```

---


### setOligoPreference

```
public void setOligoPreference(Preference oligoPreference)
```

---


### getPlasmidPreference

```
public Preference getPlasmidPreference()
```

---


### setPlasmidPreference

```
public void setPlasmidPreference(Preference plasmidPreference)
```

---


### getPlasmidPrefs

```
public java.lang.String getPlasmidPrefs()
```

---


### getStrainPreference

```
public StrainPreference getStrainPreference()
```

---


### setStrainPreference

```
public void setStrainPreference(StrainPreference strainPreference)
```

---


### getStrainPrefs

```
public java.lang.String getStrainPrefs()
```

---


### getAntibodyPreference

```
public Preference getAntibodyPreference()
```

---


### setAntibodyPreference

```
public void setAntibodyPreference(Preference antibodyPreference)
```

---


### getAntibodyPrefs

```
public java.lang.String getAntibodyPrefs()
```

---


### getOrganization

```
public Organization getOrganization()
```


---


|  |  |  |
| --- | --- | --- |
| **Overview** \| **Package** \| **Class** \| **Use** \| **Tree** \| **Deprecated** \| **Index** \| **Help** | |  |
| **PREV CLASS**   **NEXT CLASS** | **FRAMES**    **NO FRAMES**     **All Classes** |  |
| SUMMARY: NESTED \| FIELD \| CONSTR \| METHOD | DETAIL: FIELD \| CONSTR \| METHOD |  |


---
